# Latent Class Analysis of Cancer Risk Behaviors Among College Students on Guam: A Pacific Islands Cohort of College Students Study (PICCS)

**DOI:** 10.3390/ijerph22050755

**Published:** 2025-05-12

**Authors:** Aurienne Cruz, Cabrini Aguon, Michael Cajigal, Elaine C. de Leon, Reina Faye P. Evangelista, Su Bin Jin, Ella Macatugal, Gian M. Paras, Lauren Nicole G. Villanueva, Grazyna Badowski, Yvette C. Paulino

**Affiliations:** Pacific Island Partnership for Cancer Health Equity, University of Guam Cancer Research Center, Mangilao, GU 96913, USA; cruza12346@triton.uog.edu (A.C.); aguonc11869@gotritons.uog.edu (C.A.); cajigalm@triton.uog.edu (M.C.); deleone7367@triton.uog.edu (E.C.d.L.); evangelistar14100@gotritons.uog.edu (R.F.P.E.); jins@triton.uog.edu (S.B.J.); parasg@gotritons.uog.edu (G.M.P.); villanueval14580@triton.uog.edu (L.N.G.V.); gbadowski@triton.uog.edu (G.B.); paulinoy@triton.uog.edu (Y.C.P.)

**Keywords:** cancer, cancer risk factors, non-communicable diseases, Guam, latent class analysis, college students

## Abstract

This study aims to explore how cancer-related risk factors cluster among college students in Guam. Using the 2021–2022 Pacific Islands Cohort of College Students data, we conducted a latent class analysis (LCA) to organize the sample into classes based on clustering cancer risk factors, including tobacco use, binge drinking, low fruit/vegetable intake, physical inactivity, betel nut use, overweight/obesity, depression, and anxiety. Among the 577 college students surveyed, results show a high prevalence of low fruit/vegetable intake, overweight/obesity, depression, and anxiety. The LCA identified three classes, each defined by different clustering cancer risk behaviors. All classes showed high prevalence of low fruit/vegetable intake. Class 1 had the highest rates of tobacco use, betel nut use, and binge drinking. Class 2 had the highest rates of physical inactivity, depression, and anxiety. Class 3 had the lowest rates of betel nut use, overweight/obese, depression, and anxiety when compared with Classes 1 and 2. The clustering of risk behaviors highlights the need for targeted interventions and prevention strategies among Guam’s youth, aiming to address these behaviors and potentially reduce cancer risk in the region.

## 1. Introduction

The prevalence of non-communicable diseases (NCDs), such as cancer, remains high both globally and in the United States-Affiliated Pacific Islands (USAPIs). In Guam, cancer mortality rates are notably higher than in the mainland United States (U.S.), with a rate of 165.6 compared with 158.3 per 100,000 people [1]. Ninety to ninety-five percent of all cancers are primarily caused by risk factors such as environmental or lifestyle factors (e.g., tobacco, alcohol, unhealthy diets and obesity) [2]. Alcohol consumption increases the risk for cancer, with binge drinking significantly increasing the likelihood of developing alcohol-related cancers such as those of the breast, bowel, liver, mouth and throat, esophagus and stomach [3]. Sociocultural factors that promote the initiation and continuation of smoking and drinking among adults include family influence, peer influence, socioeconomic status and the availability of tobacco and alcohol products [4,5,6]. Additionally, current research has shown a positive association between binge drinking and tobacco use among adolescents and adults [4]. Contributing factors, such as high rates of tobacco consumption, betel nut use, and obesity, are prevalent in Guam—58% of adult cancers were linked to tobacco use and 32% to obesity [7]. These interconnected risk behaviors, while prevalent in Guam, reflect broader patterns observed elsewhere, highlighting the need to examine how such behaviors vary across populations. For instance, in the U.S., a latent class analysis (LCA) study explored cancer risk factors among college students, revealing that risk behaviors cluster differently by ethnicity [8]. While this study provided valuable insights into how risk behaviors are distributed within diverse populations, its findings were limited to U.S. mainland college students and did not account for cultural and environmental factors unique to island communities like Guam. Furthermore, the study did not include betel nut use, a culturally significant and highly carcinogenic behavior prevalent in Guam, nor did it address how dietary transitions toward ultra-processed foods contribute to obesity and cancer risks [9,10,11]. Further research has indicated that Guam college students in 2015 had a low frequency of fruit (77.5%) and vegetable (68%) intake; frequent fast food consumption (31.5%), physical inactivity (54.5%), obesity (22.6%), and alcohol use (44.3%); and engaged in binge drinking (24.5%), cigarette smoking (9.1%) and smokeless tobacco use (6.7%) [12]. Diets that consisted of an excessive intake of red meats and canned meats were positively associated with the development of lung, colorectal, and breast cancer [13]. On the other hand, fruit and vegetable intake showed a suggestive inverse association to cancer risk [14]. The combination of unhealthy diets and physical inactivity also promoted obesity-related health outcomes and have been associated with depression [15,16]. Research has shown that mental health issues like depression and anxiety are linked to higher incidences of lung cancer and other smoking-related cancers [17]. Depression and anxiety could also influence health behaviors, thereby increasing cancer risk. For example, depression was found to be associated with low energy and carbohydrate cravings, which contribute to decreased physical activity and poor diet [18].

Significant ethnic disparities are evident in cancer rates in Guam, with Micronesians having the highest age-adjusted cancer incidence rates at 438.0, compared with the overall Guam population rate of 279.9. Micronesians also experience the highest cancer mortality rates on the island, with lung and bronchus cancer mortality rates at 86.3—twice as high as the U.S. rate of 40.2—and cervical cancer mortality rates at 22.3, nearly ten times higher than in the U.S. Additionally, their liver cancer incidence rate is 22.0, nearly five times higher than the U.S. average of 7.8 people [1]. CHamorus also show elevated incidence and mortality rates for nasopharynx, esophagus, liver and lung cancers, with nasopharynx cancer mortality at 3.8—nineteen times higher than the U.S. rate of 0.2 [1]. Although Filipinos have lower cancer incidence rates overall, they exhibit relatively high prostate mortality rates at 23.2 when compared with the rate of 19.6 in the U.S. [1]. While traditionally linked to older populations, the increasing prevalence of cancers among younger individuals is a significant concern, as cancers are responsible for one in five adolescent deaths globally [19]. Young adults often engage in health risk behaviors, such as poor diet, physical inactivity, smoking and excessive alcohol consumption, which may lead to early-onset cancer [19]. Early intervention has long-term implications for improving quality of life, productivity and economic stability [20].

The present study aims to identify how cancer risk factors cluster across different ethnic groups within latent classes in Guam college students using LCA. By incorporating culturally relevant behaviors, such as betel nut use, and examining dietary and physical activity patterns, this study seeks to provide novel insights into the clustering of risk behaviors with Guam’s youth. The findings are expected to inform targeted interventions that address the root causes of cancer disparities in Guam, ultimately contributing to the broader understanding of how risk factors influence cancer risk.

## 2. Materials and Methods

### 2.1. Data Sources

Ethics review for the Pacific Islands Cohort of College Students (PICCS) study was obtained from the Committee on Human Research Subjects at the University of Guam (21–105). The PICCS study was developed by the University of Guam’s Health Science Program to assess cancer risk factors among college students [21]. It was pilot-tested in 2013–2014 and collected annually since 2015 from young adults (18+ years) attending the University of Guam [21]. It included surveys derived from the Guam Behavioral Risk Factor Surveillance System (BRFSS), University of Guam/University of Hawai’i Cancer Center Partnership to Advance Cancer Health Equity (PACHE), and the Children’s Health Living (CHL) program. The PICCS research study also focused on the identification of specific cancer risk behaviors. In this study, we analyzed PICCS data collected from 2021 to 2022 to determine distinct subgroups of students based on cancer risk factors of tobacco use, betel nut use, binge drinking, overweight/obesity, physical inactivity, depression, anxiety, and low fruit/vegetable intake.

### 2.2. Measures

Eight cancer risk factors were analyzed dichotomously to review whether the specific risk was present or not: tobacco use, betel nut use, binge drinking, overweight/obesity, physical inactivity, depression, anxiety, and low fruit/vegetable intake. Tobacco use was defined as being a current cigarette smoker (if the respondent reported having smoked at least 100 cigarettes in their lifetime and currently smoke “everyday” or “some days”) or current e-cigarette smokers (who reported having used e-cigarettes in their lifetime and currently use e-cigarettes “everyday” or “some days”). Current smoker and current e-cigarette smoker definitions followed the BRFSS 2022 calculated variables. Betel nut use was defined as participants who chew “everyday” and “some days.” Binge drinking was defined as having five or more drinks on one or more occasions (for males) or four or more drinks on one or more occasions (for females) in the past month, adopted by the BRFSS definition. Overweight/obesity was defined as having a body mass index (BMI; weight in kilograms divided by height in meters squared) of 25 or greater. Physical inactivity was defined as adults who did not engage in at least 150 min of moderate recreational physical activity per week, 75 min of strenuous activity per week, or an equivalent combination of moderate- and strenuous-intensity activities [22]. Depression and anxiety were assessed using the Depression Anxiety and Stress Scale-21 (DASS-21), with scores from “mild” to “extremely severe” combined to indicate the presence of these conditions. The PICCS survey collected dietary data by asking participants to report the frequency of fruit and vegetable consumption, without quantifying portion sizes or measurements. The questions were “On average, how often do you eat vegetables (including blended vegetables)” and “On average, how often do you eat fruits (including blended fruits)” with the option of choosing to give the frequency in number of times per day, week or month. We computed low fruit/vegetable intake to be defined by eating fruit or vegetables less than 5 times per day. The study population consisted of CHamorus, Filipinos, Micronesians and individuals of other ethnicities categorized as ‘Other.’ The ethnicities were chosen to represent the major ethnic groups in Guam’s population and in the Pacific region. Micronesian is defined as Pacific Islander ethnicities within Micronesia, including Chuukese, Kiribati, Kosraean, Marshallese, Palauan, Pohnpeian, Yapese and Carolinian. The ‘Other’ category includes ethnicities such as Asian (excluding Filipino), Caucasian, Hawaiian, Polynesian, Samoan, Tahitian, Tokelaun and Tongan. This last category was created due to the low number of cases of the aforementioned ethnicities. For the participant’s employment status, the category “other” was made to combine the employment categories with low counts, which include self-employed, homemaker, unable to work and retired. Unfortunately, further detail regarding the reasoning for their chosen category was not collected.

### 2.3. Statistical Analyses

Latent class analysis (LCA) is a type of finite mixture modeling used for analyzing categorical indicators [23]. It is an effective statistical method for examining cancer risk behaviors by identifying unobserved subgroups within a population based on their risk behaviors. A major advantage of LCA is its ability to reveal hidden behavioral patterns that are not easily detected through other methods. By identifying distinct clusters of behaviors, LCA helps uncover how multiple risk factors co-occur and interact, which can be crucial for designing targeted public health interventions [24]. In the present study, LCA was used to identify UOG students’ subgroups with similar cancer risk factor profiles based on the patterns in the data. Various class sizes, ranging from two to five, were compared to determine the best-fit model. The Akaike information criterion (AIC) served as the primary criterion for model selection as opposed to the Bayesian information criterion (BIC), due to the BIC’s tendency to penalize larger sample sizes. In addition, diagnostic statistics, such as entropy and the average latent class posterior probability (ALCPP), were evaluated for model accuracy to guide final model selection. Entropy measures how accurately the model defines classes, where values closer to 1 demonstrate higher classification accuracy and values below 0.6 indicate poor classification [24]. ALCPP indicates the average probability that the model accurately predicts an individual’s class membership. LCA was conducted using the poLCA package [25] in RStudio version 4.2 software (Available online: https://r-studio.software.informer.com/4.2/#google_vignette) (accessed on 15 September 2024). Five different latent class analysis (LCA) models were compared using fit criteria, including the BIC, AIC, likelihood ratio (LR), and chi-square statistics (X^2^), as well as diagnostic measures such as entropy and ALCPP.

The three-class model had the lowest AIC value (4576.78), indicating the best fit among the models. Although the entropy value for the three-class model was slightly lower (0.68) when compared with the two-class, four-class, and five-class models (all with entropy = 0.7), it was still deemed acceptable as it provided sufficient class separation. As model selection does not rely on entropy alone, the three-class model offers a reasonable balance between defined classes and interpretability. Additionally, the ALCPP for the three-class model was 0.89, which exceeded the recommended threshold of 0.8 [26].

Based on these criteria, the three-class model was selected due to its superior AIC performance, despite its slightly lower entropy compared with some of the other models. This model also avoided the issue of very small class sizes, which were evident in the four-class and five-class models. The chi-square tests were used to evaluate the differences in the prevalence of risk factors across latent classes. For risk factors with an expected cell count of less than 5, bootstrapping with 1000 resamples from the original dataset was applied to ensure robust estimates. Additionally, subgroup analyses were conducted within each latent class, using chi-square tests to determine whether the distribution of risk factors differed across racial/ethnic groups. SPSS version 29 (IBM, Armonk, NY, USA) was used for the tests, with a significance level of 0.05 applied to all analyses. Additionally, all tables were computed using valid percentages.

## 3. Results

Participant characteristics are summarized in Table 1. Of the total 577 participants, approximately two-thirds (64.2%) were female. Most participants identified as Filipino (49.1%), followed by CHamoru (23.9%), Micronesian (17.6%), and Other (9.4%). Three participants did not indicate their ethnicity. Juniors made up the largest group by academic level (30.6%), while graduate students represented the smallest (5.8%).

Low fruit/vegetable intake was the most prevalent cancer risk factor (91.3%), followed by depression (61.1%), and anxiety (57.1%). About a third of participants reported physical inactivity (30.9%) and half reported overweight/obesity (50.4%). A quarter of participants reported binge drinking (24.9%). Meanwhile, tobacco (23.4%) and betel nut use (5.3%) were the lowest reported cancer risk factors. Across all ethnic groups, Micronesians had the highest rates for tobacco use (38.4%), betel nut use (24.7%), physical inactivity (42.3%), overweight/obesity (62.8%), depression (67.3%) and low fruit and vegetable intake (93.8%). Meanwhile, Filipinos experienced the greatest anxiety (61.0%) and Others experienced the greatest binge drinking (32.5%) (Table 2).

The results of the LCA identified a three-class model. The results of the class criteria evaluation are presented in Table 3. Class 1, Class 2 and Class 3 consisted of 10.1%, 38.3% and 51.6% of the total sample (Table 4a), respectively. A low fruit/vegetable intake and overweight/obesity were prevalent among students across all classes. Class 1 showed the highest rates of tobacco use (100.0%), binge drinking (97.6%), betel nut use (20.7%) and overweight/obesity (58.2%). Class 2 showed the highest rates of depression (100%), anxiety (100%) and physical inactivity (39.6%). Class 3 exhibited the lowest rates of betel nut use (3.1%), overweight/obesity (46%), depression (28.7%) and anxiety (20.1%). The chi-square test showed significant differences for all risk factors by latent classes except for overweight/obesity, which was marginally significant, and low fruit/vegetable intake—the *p*-values were 0.061 and 0.886, respectively.

Data analysis revealed some differences in cancer risk behavior clustering among ethnicities (Table 4b). Filipinos and “Other” ethnic groups showed no prevalence of betel nut use in any class.

- **Class 1**: Tobacco use, betel nut use, binge drinking, and overweight/obesity showed no significant differences across ethnic groups.
- **Class 2**: Significant differences between ethnic groups were observed for tobacco use (*p* < 0.001), betel nut use (*p* < 0.001), overweight/obesity (*p* = 0.043), and physical inactivity (*p* = 0.034). Tobacco use ranged from 4.1% among Filipinos to 24.4% among CHamorus, while betel nut use was highest among Micronesians (21.6%) and lowest among Filipinos (0%). Overweight/obesity prevalence ranged from 41.2% among the “Other” group to 68.4% among CHamorus. Physical inactivity was most prevalent among Micronesians (55.3%) and least among Filipinos (41.3%). Depression, anxiety and fruit/vegetable intake did not differ significantly across ethnic groups within Class 2.
- **Class 3**: Significant differences were observed in overweight/obesity, with Micronesians having the highest rates (64.3%) and Filipinos the lowest (38.9%). Class 3 also showed a significant difference in the prevalence of tobacco use (*p* = 0.025), where Micronesians had the highest rates (38.4%) and Filipinos had the lowest (12.9%).

## 4. Discussion

This study identifies three distinct cancer risk behavior patterns among Guam’s college students. Class 1 had the highest rates for tobacco use, betel nut use, binge drinking, and overweight/obesity. All ethnicities within Class 1 had the same rate of tobacco use (100.0). Micronesians had the highest rate of betel nut use (47.4), followed by CHamorus (15.4). CHamorus, Filipinos, and Others had the same rate of binge drinking (100.0). These behaviors are expected to cluster because individuals who chew betel nut often engage in smoking and drinking behaviors as well [27]. Betel nut acts as a mild stimulant, and its use is associated with an increased likelihood of tobacco consumption [28]. Combined use can elevate health risks, notably in increasing the risk for cancers of the mouth, lungs and esophagus [29]. Furthermore, this practice is common among both adolescents and adults, driven by social and cultural factors that promote this behavior [30]. In a study involving Pacific Islanders from Guam, Saipan, Chuuk and Palau, 45% reported chewing betel nut with tobacco during their last chewing session [30]. Alcohol consumption is typically paired with these behaviors, which is observable in Class 1 [31]. The concurrent use of alcohol and cigarettes among adolescents is driven by common social and developmental factors. Adolescents who drink are more likely to smoke, with each behavior reinforcing the other. Together, drinking and smoking act as “gateway” behaviors, often leading to increased risk of additional substance use [32,33].

Class 2 exhibited the highest rates of physical inactivity, depression and anxiety. Micronesians had the highest rate of physical inactivity (55.3), followed by Filipinos (41.3), Other (27.8) and CHamorus (25.0). All ethnicities in Class 2 had the same rate of depression and anxiety (100.0). The clustering of these behaviors is expected as low physical activity levels are commonly linked to a heightened risk of depression and anxiety [34]. Supporting this, a meta-analysis of 115,540 children and adolescents across 12 countries found a strong association between meeting exercise guidelines and positive mental health outcomes [35]. Additionally, physical activity has consistently been associated with improved mental health, suggesting that inactivity may correlate with poorer mental health outcomes. The link between physical inactivity, depression and anxiety is further supported by biological and psychological mechanisms [16,36]. Biologically, inactivity can lead to increased inflammation, reduced neurogenesis and decreased neuroplasticity, all of which contribute to the development of mental health disorders [34]. Psychologically, physical inactivity negatively affects self-esteem and feelings of physical competence, further increasing the risk of depression and anxiety [34]. In contrast, Class 3 had the lowest rates of betel nut use, overweight/obesity, depression and anxiety, suggesting protective behavioral and lifestyle factors that could lower cancer risk. This clustering is consistent with research highlighting a positive and statistically significant association between obesity and mental health issues, where reduced obesity rates are linked with improved psychological well-being [37]. Class 3 does not have any defining risk behaviors, making it the relatively healthier sample of the three. Participants in this class had the lowest rates of physical inactivity, depression, anxiety, betel nut use and obesity. Class 3 had a higher overall rate of tobacco use than class 2. However, Class 2 had a higher rate of binge drinking than Class 3. Class 3 had the highest rate of fruit and vegetable intake.

Overall, low fruit and vegetable intake emerged as the most prevalent cancer risk factor among Guam’s college students, with 91.3% reporting insufficient consumption, followed by depression (61.1%) and anxiety (57.1%). Dietary recommendations by the World Health Organization and Food and Agriculture of the United Nations suggest at least five servings of fruit and vegetables daily, with an exclusion of starchy vegetables [38]. Studies suggest that low intake of fruits and vegetables may contribute to cancer risk due to reduced intake of essential nutrients that combat oxidative stress and inflammation. For example, fruits and vegetables are rich in essential nutrients such as vitamins, fiber and bioactive compounds like antioxidants, which help combat DNA damage from free radicals, a known factor in cancer development [13]. Sufficient intake of fruits and certain vegetables during adolescence can be linked to a significantly lower risk of cancer, suggesting that early dietary habits may have long-term protective effects against cancer [38]. For example, in a cross-sectional study assessing cancer risk and fruit and vegetable intake, participants who met recommendations of five or more servings of fruit and vegetable per day had 87% lower odds of reporting for cancers [39]. Furthermore, a meta-analysis of 13 cohort studies found that the summary relative risk for any cancer incidence was 0.96 (95% confidence interval [CI]: 0.95–0.97) for every 200 g/day increase in fruit and vegetable intake [40]. Results of low fruit and vegetable intake in this study may be due to Guam’s reliance on imported food, which has made fresh fruits and vegetables particularly expensive, with costs driven by high shipping fees and regulatory constraints like the Jones Act [41]. Grocery store prices for produce are often significantly higher than at local flea markets, where residents can find more affordable options by purchasing locally grown items early in the day. However, even these local markets face challenges in providing consistent access to affordable fresh produce due to limited agricultural output and competition from cheaper imported goods [42]. These high costs contribute to limited fruit and vegetable consumption among residents who may struggle to afford these healthier options [43]. Mental health challenges, such as depression and anxiety, also influence cancer risk. Depression may raise the likelihood of certain cancers, such as lung, gastrointestinal and breast cancers, potentially due to associated behaviors like smoking and the inflammatory effects of chronic stress [44]. When depression or anxiety coexist with other risk factors, such as substance use, the cumulative risk for cancer appears higher, highlighting the influence of both behavioral and biological factors [17].

Our results also indicate that Guam college students have higher rates of several cancer risk behaviors, such as overweight/obesity (50.4% vs. 36.5%), anxiety (57.1% vs. 27.4%), and depression (61.1% vs. 21.7%), when compared with their U.S. counterparts [45]. These findings are consistent with previous studies that found students on Guam to have higher depression and anxiety compared with students in the mainland U.S. [46]. Tobacco use rates in Guam also exceed those of the U.S. (23.4% vs. 19.2%), which aligns with higher smoking initiation in Guam youth [29]. Furthermore, the results are consistent with BRFSS (2015) data, which indicates higher percentages of binge drinking among college students compared with adults in Guam. Interestingly, physical inactivity rates are the same between Guam and the U.S. (30.9%) [45]. Extending LCA to different ethnicities among Guam’s college students revealed diverse patterns of cancer risk behaviors. Tobacco use was highest amongst Micronesians (Table 4b). This is consistent with the findings of the Youth Risk Behavior Survey in 2013, where Micronesian youth were found to have significantly higher rates of smoking in Guam compared with U.S. averages, with 27.2% being current smokers (vs. 15.7% in the U.S.) and 39.7% using smokeless tobacco (vs. 8.8% in the U.S.). They also have the highest smoking rates among all ethnic groups in Guam [47]. CHamoru students exhibit the highest rates of overweight and obesity across all classes, followed by Micronesians and Filipinos. This pattern may reflect the global trend of rising obesity linked to dietary shifts toward low-nutrient, processed foods [48]. Additionally, limited access to affordable, nutritious food in indigenous and marginalized communities could contribute to these disparities [49,50]. The elevated rates of betel nut use among Micronesian students in Classes 1 and 2 may be related to its cultural relevance in Pacific Island communities. Betel nut use is typically high among Micronesians due to its strong cultural acceptance, social integration, and easy accessibility, particularly amongst adolescents [51]. Given that cancer is a multimodal disease, there may be other factors influencing the clustering of these behaviors. The clustering of cancer-related risk factors may also be influenced by the high prevalence of other non-communicable diseases (NCDs), such as diabetes. Diabetes, which shares behavioral and metabolic risk factors like obesity, poor diet and physical inactivity, has been a significant public health concern in Guam and the broader Pacific region. The regional declaration of an NCD emergency in 2010 underscores the interconnectedness of these risk factors and their contribution to chronic disease burdens, including cancer [21].

This study has several strengths, including its use of LCA to comprehensively understand how cancer risk behaviors cluster among Guam college students. LCA enhances the understanding of cancer risk profiles by grouping individuals into latent classes based on their behaviors, thus offering insights into how these behaviors relate to cancer outcomes [8]. Furthermore, the use of the poLCA package in RStudio version 4.2 software validates the analysis, supported by a robust sample size. The use of the PICCS data strengthens the study by providing a dataset specifically designed to assess health risk factors among Guam’s college student population. Furthermore, the inclusion of various ethnicities within Guam’s college student population identifies diverse patterns of health behaviors across different cultural backgrounds. However, this study does not account for genetic variations that may contribute to cancer susceptibility across ethnic groups. Genetic predisposition can influence cancer risk independently or in conjunction with lifestyle behaviors, and future research should integrate genetic and behavioral data to better understand these interactions [52,53].

This study also has some key limitations. Responses were self-reported, which is prone to reporting bias. Additionally, LCA categorizes individuals based on probabilities, which means that accurate class assignment is not always assured [54]. This approach restricts the ability to determine exact counts or percentages within each class. The complexity involved in model specification and the challenge of determining the optimal number of latent classes can impact the reliability and interpretability of results [55]. LCA relies on assumptions such as the mutual exclusivity and exhaustiveness of classes. Deviations from these assumptions can lead to misleading conclusions [24]. In this case, the ALCPP value of 0.89 indicates compliance with the assumption. We also considered entropy as a supplementary measure to evaluate classification quality. Entropy values closer to 1.0 indicate higher accuracy in class membership, with a value of 0.7 being acceptable for distinguishing classes. “Naming fallacy” may also occur when the name assigned to a class misrepresents its defining characteristics. Despite these challenges, LCA remains a valuable tool for exploring patterns of cancer risk behaviors that may aid in informing prevention strategies.

These findings underscore the critical need for early intervention to mitigate cancer risk in young adults in Guam, as high-risk behaviors established during college years can persist into adulthood and exacerbate future health issues. Targeted interventions should focus on enhancing physical activity, mental health support, and access to nutritious foods among college students, particularly for those in high-risk classes identified through our LCA. Further research should explore the efficacy of culturally tailored health interventions for Guam’s ethnic groups. For example, the high rates of tobacco and betel nut use among Micronesian students suggest the need for culturally sensitive smoking cessation programs that address the social and cultural dimensions of these behaviors. One such effort is the Betel Nut Intervention Trial (BENIT) in Guam, which has demonstrated success in promoting cessation through tailored interventions that consider the cultural significance of betel nut use in Micronesian communities [56]. Future research might also consider developing new culturally tailored health interventions or education sessions. The clustering of physical inactivity, depression, and anxiety in Class 2 indicates that mental health and lifestyle interventions could be effective in reducing co-occurring risk factors. Future studies may also examine the impact of socioeconomic factors, such as access to healthy food and safe recreational spaces, on risk behavior patterns across ethnic groups. Expanding the sample to include non-college populations would improve the generalizability of these findings and allow for comparisons across different educational and socioeconomic backgrounds. Additionally, longitudinal research could help to determine whether early behavioral interventions among college students yield long-term reductions in cancer prevalence in adulthood. The lack of sufficient literature applying LCA to Guam’s population emphasizes the need for more localized research to better understand and address the underlying causes of these behaviors.

## 5. Conclusions

This study conducted a latent class analysis (LCA) to explore the clustering of cancer risk behaviors among college students in Guam. The results identify three distinct classes characterized by unique combinations of behaviors: Class 1 showed high levels of tobacco use, binge drinking, and betel nut use; Class 2 was defined by high rates of physical inactivity, depression and anxiety; while Class 3 exhibited lower overall prevalence of risk factors and represented the healthiest group. Across all classes, low fruit and vegetable intake emerged as the most prevalent cancer risk factor, highlighting a critical area for intervention. These findings underscore the need for targeted interventions to address the specific clusters of behaviors associated with increased cancer risk. Interventions should focus on the promotion of healthier dietary habits, enhancing mental health support, and addressing the sociocultural factors contributing to substance use. Longitudinal research and expanded population studies are recommended to assess the long-term impact of early behavioral interventions on cancer outcomes in Guam and other Pacific Islander populations. This research contributes to the understanding of cancer risk in young adults, emphasizing the importance of public health strategies by which to address clustering risk factors and foster healthier communities in the Pacific region. Such tailored interventions may offer a more impactful approach to cancer prevention and help reduce risk behaviors that may persist into adulthood.

## Figures and Tables

**Table 1:** PICCS participants characteristics of the full sample by sex—University of Guam, years 2021–2022.

	All	Male	Female
	*N* (%)	*N* (%)	*N* (%)
	577	202 (35.8)	362 (64.2)
**Age group quartiles**			
18–19	135 (23.9)	39 (19.3)	93 (26.1)
20–21	150 (26.5)	57 (28.2)	91 (25.6)
22–23	142 (25.1)	48 (23.8)	92 (25.8)
24+	138 (24.4)	58 (28.7)	80 (22.5)
**Body mass index (BMI)**			
Underweight (<18.5)	29 (5.3)	6 (3.1)	22 (6.5)
Normal weight (18.5–24.9)	241 (44.3)	77 (39.3)	162 (47.6)
Overweight (25–29.9)	132 (24.3)	59 (30.1)	72 (21.2)
Obese (≥30)	142 (26.1)	54 (27.6)	84 (25.7)
**Employment**			
Employed	246 (59.6)	93 (60.0)	146 (59.1)
Not employed	108 (26.2)	41 (26.5)	64 (25.9)
Other *	59 (14.3)	21 (13.5)	37 (15.0)
**Ethnicity**			
CHamoru	137 (23.9)	37 (18.3)	98 (27.3)
Filipino	282 (49.1)	110 (54.5)	165 (46.0)
Micronesian	101 (17.6)	33 (16.3)	67 (18.7)
Other **	54 (9.4)	22 (10.9)	29 (8.1)
**Health insurance**			
No	159 (30.5)	46 (26.1)	108 (32.0)
Yes	363 (69.5)	130 (73.9)	229 (68.0)
**Student level**			
Freshman	103 (18.1)	29 (14.6)	72 (20.2)
Sophomore	99 (17.4)	35 (17.6)	62 (17.4)
Junior	174 (30.6)	70 (35.2)	101 (28.3)
Senior	159 (28.0)	55 (27.6)	100 (28.0)
Graduate	33 (5.8)	10 (5.0)	22 (6.2)
**Years in college**			
1 year or less	60 (11.2)	20 (10.4)	39 (11.7)
2 years	79 (14.8)	25 (13.0)	51 (15.3)
3 years	113 (21.1)	46 (24.0)	65 (19.5)
4 years	118 (22.1)	42 (21.9)	74 (22.2)
5 or more years	165 (30.8)	59 (30.7)	104 (31.2)

“All” reflects the full sample (*N* = 577). Other * = self-employed, homemaker, unable to work, retired. Other ** = Asian, Caucasian, other Native Hawaiians/Pacific Islanders. Tables reflect valid percentages. Counts may not add up to *N* = 577 due to missing data by variable. Number of missing values: Gender = 13, Age groups = 12, BMI = 33, employment status = 3, health insurance status = 55, student level = 9, college years = 42.

**Table 2:** Descriptive statistics and rates of cancer risk behaviors stratified by total and ethnicity.

		Ethnicity (*N* = 574)			
	All	CHamoru	Filipino	Micronesian	Other **
	*N* (%)	*N* (%)	*N* (%)	*N* (%)	*N* = 67
Tobacco use	134 (23.4)	39 (28.5)	40 (14.3)	38 (38.4)	16 (23.9)
Betelnut use	30 (5.3)	5 (3.8)	0 (0)	24 (24.7)	0 (0)
Binge drinking	122 (24.9)	29 (24.0)	58 (23.0)	22 (29.3)	15 (27.8)
Overweight/obesity	274 (50.4)	74 (56.1)	116 (43.8)	59 (62.8)	28 (47.5)
Physical inactivity	174 (30.9)	31 (23.1)	93 (33.5)	41 (42.3)	10 (15.2)
Depression	349 (61.1)	75 (55.1)	173 (62.5)	68 (67.3)	38 (56.7)
Anxiety	326 (57.1)	67 (49.3)	169 (61.0)	59 (58.4)	35 (52.2)
Low fruit/vegetable Intake	441 (91.3)	94 (87.9)	230 (93.5)	75 (93.8)	51 (87.9)

“All” reflects the full sample (*N* = 577). Other ** = Asian, Caucasian, other Native Hawaiians/Pacific Islanders. Tables reflect valid percentages. Counts may not add up to the total due to missing data by variable.

**Table 3:** Class criteria characteristics.

**Model Fit Criteria**				
**Classes**	**BIC**	**AIC**	**LR**	**X^2^**
1	4736.57	4701.71	277.48	372.79
2	**4678.87**	4604.79	188.49	263.17
3	4690.08	**4576.78**	155.29	189.37
4	4730.92	4578.40	144.39	183.38
5	4772.58	4580.83	132.68	161.98
**Diagnostic Criteria**				
**Classes**	**Smallest Class Count (n)**	**Smallest Class Size (%)**	**Entropy**	**ALCPP**
1	577	1	-	-
2	217	0.38	0.7	0.92
3	64	0.12	0.68	0.89
4	12	0.11	0.7	0.88
5	13	0.02	0.7	0.86

Note: Bold text indicates the model met the fit criteria. BIC = Bayesian information criterion; AIC = Akaike information criterion; LR = log ratio; X^2^ = chi-square test; ALCPP = average latent class posterior probability.

**Table 4:** (**a**) Prevalence of risk behaviors within latent classes (*N* = 577). (**b**) Prevalence of risk factors within latent classes by ethnicity (*N* = 574).

(**a**)								
**Class (% of Total Sample)**	**Tobacco Use**	**Betel Nut Use**	**Binge Drinking**	**Overweight/Obese**	**Physical Inactivity**	**Depression**	**Anxiety**	**Fruit/Veg Intake**
Class 1 (10.1)	100.0	20.7	97.6	58.2	16.1	77.2	80.7	91.8
Class 2 (38.3)	10.0	4.1	14.0	54.8	39.6	100.0	100.0	92.0
Class 3 (51.6)	18.4	3.1	21.3	45.6	27.2	28.7	20.1	90.7
*p*	<0.001 *	<0.001 *	<0.001 *	0.061	<0.001 *	<0.001 *	<0.001 *	0.886
(**b**)								
	**Tobacco Use**	**BetelNut Use**	**Binge Drinking**	**Overweight/Obese**	**Physical Inactivity**	**Depression**	**Anxiety**	**Fruit/Veg Intake**
**Class 1**								
CHamoru (*N* = 13)	100	15.4	100	84.6	7.7	61.5	61.5	100
Filipino (*N* = 17)	100	0	100	46.7	17.6	81.3	93.8	85.7
Micronesian (*N* = 19)	100	47.4	91.7	50	23.5	84.2	84.2	100
Other (*N* = 8) **	100	0	100	50	0	75	75	87.5
*p*	NA	0.101 ^b^	0.488 ^b^	0.070 ^b^	0.480 ^b^	0.203 ^b^	0.238 ^b^	0.289
**Class 2**								
CHamoru (*N* = 41)	24.4	2.4	12.8	68.4	25	100	100	85.3
Filipino (*N* = 123)	4.1	0	14.3	48.7	41.3	100	100	92.7
Micronesian (*N* = 38)	13.2	21.6	13.3	67.6	55.3	100	100	96.7
Other (*N* = 19) **	10.5	0	16.7	41.2	27.8	100	100	92.9
*p*	<0.001 ^b^	<0.001 ^b^	0.849 ^b^	0.043	0.034	NA	NA	0.415 ^b^
**Class 3**								
CHamoru (*N* = 83)	19.3	2.5	17.1	45.7	24.7	31.7	22	87.1
Filipino (*N* = 142)	12.9	0	22.8	38.9	28.6	26.8	22.5	95.1
Micronesian (*N* = 44)	33.3	17.1	21.2	64.3	38.1	31.8	11.4	88.6
Other (*N* = 27) **	22.2	0	26.1	46.2	11.1	25.9	18.5	80.8
*p*	0.025	<0.001 ^b^	0.75	0.041	0.092	0.824	0.429	0.051 ^b^

^b^ Bootstrapped chi square due to expected cell counts <5. NA indicates no statistical tests were conducted. * *p* < 0.05. Other ** includes Asian, Caucasian, other Native Hawaiians/Pacific Islanders. Tables reflect valid percentages.

## Data Availability

The PICCS data may be accessed by contacting Yvette C. Paulino.

## Abbreviations

The following abbreviations are used in this manuscript:

NCD	Noncommunicable disease
USAPI	United States-Affiliated Pacific Islands
LCA	Latent class analysis
PICCS	Pacific Islands Cohort of College Students Study
AIC	Akaike information criterion
ALCPP	Average latent class posterior probability
BIC	Bayesian information criterion
